# Airborne particulate matter (PM_2.5_) triggers ocular hypertension and glaucoma through pyroptosis

**DOI:** 10.1186/s12989-021-00403-4

**Published:** 2021-03-04

**Authors:** Liping Li, Chao Xing, Ji Zhou, Liangliang Niu, Bin Luo, Maomao Song, Jingping Niu, Ye Ruan, Xinghuai Sun, Yuan Lei

**Affiliations:** 1 Department of Ophthalmology & Visual Science, Eye Institute, Eye & ENT Hospital, Shanghai Medical College, Fudan University, Shanghai, 200031 China; 2 Experimental Research Center, Eye & ENT Hospital, Shanghai Medical College, Fudan University, Shanghai, 200031 China; 3 Shanghai Key Laboratory of Meteorology and Health, Shanghai, 200030 China; 4 Institute of Occupational Health and Environmental Health, School of Public Health, Lanzhou University, Lanzhou, 730000 Gansu China; 5 Shanghai Key Laboratory of Meteorology and Health, Shanghai Meteorological Bureau, Shanghai, China; 6 NHC Key Laboratory of Myopia, Chinese Academy of Medical Sciences (Fudan University), and Shanghai Key Laboratory of Visual Impairment and Restoration (Fudan University), Shanghai, 200031 China; 7 State Key Laboratory of Medical Neurobiology and MOE Frontiers Center for Brain Science, Institutes of Brain Science, Fudan University, Shanghai, 200032 China

**Keywords:** PM_2.5_, NLRP3, Intraocular pressure, Human trabecular meshwork cell, Pyroptosis

## Abstract

**Background:**

Particulate matter (PM) is strongly linked to human health and has detrimental effects on the eye. Studies have, however, focused on the ocular surface, with limited research on the impact of PM_2.5_ on intraocular pressure (IOP).

**Methods:**

To investigate the impact of PM_2.5_ on IOP and the associated mechanism, C57BL/6 mouse eyes were topically exposed to a PM_2.5_ suspension for 3 months, and human trabecular meshwork (HTM) cells were subjected to various PM_2.5_ concentrations in vitro. Cell viability, NLRP3/caspase-1, IL-1β, and GSDMD expression, reactive oxygen species (ROS) production and cell contractility were measured by western blot, ELISA, cell counting kit-8, ROS assay kit or a cell contractility assay. ROS scavenger N-acetyl-L-cysteine (NAC) and caspase-1 inhibitor VX-765 were used to intervene in PM_2.5_-induced damages.

**Results:**

The results revealed that the IOP increased gradually after PM_2.5_ exposure, and upregulations of the NLRP3 inflammasome, caspase-1, IL-1β, and GSDMD protein levels were observed in outflow tissues. PM_2.5_ exposure decreased HTM cell viability and affected contraction. Furthermore, elevated ROS levels were observed as well as an activation of the NLRP3 inflammasome and downstream inflammatory factors caspase-1 and IL-1β. NAC improved HTM cell viability, inhibited the activation of the NLRP3 inflammasome axis, and HTM cell contraction by scavenging ROS. VX-765 showed similar protection against the PM_2.5_ induced adverse effects.

**Conclusion:**

This study ﻿provides novel evidence that PM_2.5_ has a direct toxic effect on intraocular tissues and may contribute to the initiation and development of ocular hypertension and glaucoma. This occurs as a result of increased oxidative stress and the subsequent induction of NLRP3 inflammasome mediated pyroptosis in trabecular meshwork cells.

**Supplementary Information:**

The online version contains supplementary material available at 10.1186/s12989-021-00403-4.

## Highlights

- PM_2.5_ exposure increases intraocular pressure accompanied by intraocular tissue pyroptosis
- PM_2.5_ triggers oxidative stress which activates pyroptosis in human trabecular meshwork cells
- NLRP3 inflammasome mediates PM_2.5_ induced trabecular meshwork pyroptosis

## Introduction

Epidemiological and experimental studies suggest that particulate matter (PM), especially PM_2.5_ (aerodynamic diameter ≤ 2.5 μm), is strongly associated with respiratory, cardiovascular, metabolic, and even emotional disorders [1]. Although the eyes are in direct contact with the external environment, studies on the impact of PM_2.5_ on ocular health remain scarce [2]. According to previous studies, PM causes ophthalmic diseases such as conjunctivitis, keratitis, and ﻿dry eye syndrome [3–6]. However, these PM-associated studies focused on the ocular surface, with little consideration of the particles’ potential to penetrate the human cornea and affect tissues within the eye, and their involvement in the initiation and development of the intraocular diseases.

The recent increase in air pollution may be an important cause of, for example, glaucoma [7]. An epidemiological study, for the first time, presented a relationship between long-term air pollution and intraocular pressure (IOP) elevation [7]. Other studies have demonstrated the association between PM_2.5_ exposure and glaucoma, without any link to IOP elevation, but through neurotoxicity and vascular dysfunction in the retina [8, 9]. Therefore, conducting investigations to elucidate the link between PM_2.5_ and glaucoma as well as the associated underlying mechanisms is of relevance.

The production and outflow of aqueous humor dynamically maintain IOP. Outflow resistance is reported in the juxtacanalicular tissue region, where adjacent trabecular meshwork (TM) and Schlemm’s canal endothelial cells regulate aqueous humor outflow [10]. Cellular or extracellular stimuli such as oxidative stress can damage the TM, causing inflammation and cell death, thereby causing IOP elevation and, eventually, glaucoma [11, 12].

The current study aimed to investigate the association between PM_2.5_ and ocular hypertension and to elucidate the mechanisms underlying this relationship. This study was incentivized by the unintentional discovery that topical application of fluorescent mock PM_2.5_ to the eye caused deposition in outflow tissues including the iris, ciliary body, and TM. These tissues are vital for regulating aqueous humor outflow resistance and IOP [10]. The current findings suggest that, apart from its involvement in ocular surface disease, PM_2.5_ pollution also affects tissues inside the eye, possibly participating in the development of intraocular diseases such as glaucoma.

## Results

### The size distribution of the PM

**Fig. 1:**
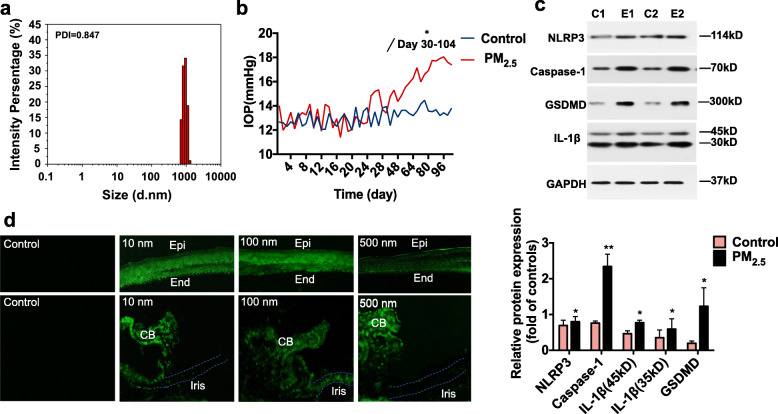
**a** Particle size distribution analyzed by dynamic light-scattering. PM_2.5_ suspension was analyzed by dynamic light-scattering for three times and the average sizes of PM is 876 nm. **b** PM_2.5_ exposure induced ocular hypertension in C57BL/6 mice. PM_2.5_ suspension was applied topically to the mouse eyes. The intraocular pressure (IOP) was measured without anesthesia by rebound tonometry. IOP was elevated gradually in PM_2.5_-treated mouse eyes compared with the controls (*n* ≥ 6, *P < 0.05, paired sample t-test or Mann-Whitney U test). **c** PM_2.5_ increased the expressions of NLRP3 inflammasome-related proteins in mouse eye outflow tissues. Relative protein expressions of NLRP3 (*n* = 3), caspase-1 (*n* = 4), GSDMD (*n* = 3) and IL-1β (*n* = 3) were determined by western blot and showed in A-D. *P < 0.05, independent sample t-test. Date are represented as the mean ± standard deviation. C, PBS-treated control; E, PM_2.5_ exposure. **d** Mock PM_2.5_ particles penetrated the mouse cornea and deposited in intraocular tissues. Mock PM_2.5_ particles with diameters ranging from 10 nm to 500 nm passed through the cornea, entered the anterior chamber and finally deposited on the outflow tissue. The dotted line shows the outline of the iris. Epi, cornea epithelium; end, cornea endothelium; CB, ciliary body

The dynamic light-scattering of the PM suspension was analyzed and the result revealed PM with sizes ranging between 712 nm and 1280 nm. 99.95% of the particles were less than 1280 μm (Fig. 1a).

### PM_2.5_ exposure induced ocular hypertension in C57BL/6 mice

Elevated IOP values were observed in mouse eyes exposed to PM_2.5_ from day 25 to 104. During this period, the average IOP elevation was 2.6 mmHg, with a maximum of 4.7 mmHg observed on day 92 (*n* = 7, *P* < 0.05, Fig. 1b). From day 1 to day 24, IOP values for both eyes (treated and controls) were similar. On day 25, IOP values of PM_2.5_-treated eyes significantly increased relative to those of phosphate-buffered saline (PBS)-treated eyes (14.8 ± 0.6 mmHg, *n* = 10 vs 12.3 ± 0.8 mmHg, *n* = 7, *P* < 0.05). Elevated IOP was sustained for 3 days, followed by 2 days of normalization. Thereafter, IOP values increased again and remained consistently higher compared to the values of control eyes. IOP elevation was steadily significant from day 30 onwards, with IOP values of 14.9 ± 1.4 mmHg and 12.4 ± 1.1 mmHg for PM_2.5_-treated eyes and PBS-treated eyes, respectively (PBS treated, *n* = 10; PM_2.5_-treated, *n* = 10; *P* < 0.05). Our data demonstrate that PM_2.5_ exposure adversely affected IOP, thus representing a risk factor for glaucoma.

### Characteristic features of pyroptosis in outflow tissues following PM_2.5_ exposure

To understand the underlying mechanism of PM_2.5_-related ocular hypertension, we measured the levels of proteins associated with the classic pyroptosis pathway (NLRP3/caspase-1/IL-1β/GSDMD) in aqueous humor outflow tissue (Fig. 1c). NLRP3 expression increased 1.2-fold in outflow tissue of PM_2.5-_treated eyes compared to that of control eyes (*n* = 3, *P* < 0.05). Furthermore, levels of the NLRP3 downstream proteins caspase-1 and GSDMD increased 3.1- and 6.3-fold, respectively (*n* = 4, *P* < 0.05). In addition, total IL-1β was 1.7 times higher in outflow tissue from PM_2.5_-treated eyes compared to contralateral controls, whereas cleaved IL-1β increased 5.5-fold (*n* = 3, *P* < 0.05). These observations suggested that PM_2.5_ caused tissue injury via pyroptosis of cells in outflow tissue of the eye.

To understand PM_2.5_ entry into the anterior chamber, fluorescent mock PM_2.5_ tracers were topically applied to the eye. Particles with diameters from 10 to 500 nm passed through the cornea into the anterior chamber and were mainly deposited in outflow tissue, with ciliary body deposition being the most pronounced (Fig. 1d). Fluorescent tracers distributions revealed that some PM_2.5_ crossed the cornea and entered the eye, thereby affecting intraocular function.

### PM_2.5_ exposure triggered pyroptosis in HTM cells

**Fig. 2:**
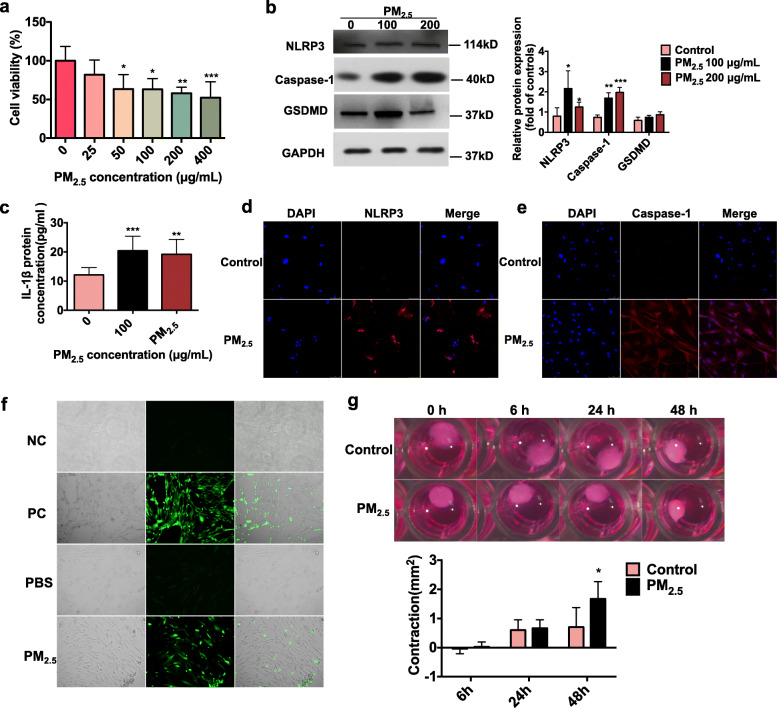
**a** The adverse effect of PM_2.5_ exposure on HTM cell viability. Cell viability were analyzed by CCK-8 cell viability assay. The human trabecular meshwork cells were treated with different concentrations of PM_2.5_ (0 μg/mL, 25 μg/mL, 25 μg/mL, 50 μg/mL, 100 μg/mL, 200 μg/mL, or 400 μg/mL) for 48 h. (*n* = 5 cell lines, *P < 0.05, **P < 0.01, ***P < 0.001, by one-way ANOVA, compared with the control). Date are represented as the mean ± standard deviation. **b**, **c** Expressions of NLRP3 inflammasome-related proteins in PM_2.5_-treated TM cells. After HTM cells were treated by PM_2.5_ (100 μg/mL and 200 μg/mL), relative protein expressions of NLRP3 (*n* = 4 cell lines), caspase-1 (*n* = 3 cell lines), and GSDMD (*n* = 3 cell lines) determined by western blot and IL-1β by ELISA (*n* = 4 cell lines). *P < 0.05, one-way ANOVA. **d**, **e** Immunofluorescence examination suggests increased expressions of NLRP3 and caspase-1 in HTM cells after PM_2.5_ exposure. Representative immunofluorescence images of the punctate staining of NLRP3 (D) and caspase-1 (E) in HTM cells treated with the vehicle control or PM_2.5_ 100 μg/mL for 48 h. Blue, DAPI; red, NLRP3 or caspase-1. Magnification, 20X. **f**. ROS production in PM_2.5_-treated HTM cells. ROS fluorescence staining images of Rosup negative control (NC, 50 μg/mL), Rosup positive control (PC, 500 μg/mL, PBS control group (Control) and PM_2.5_ 100 μg/mL group (PM_2.5_). ROS were detected by the reactive oxygen species assay kit. In each panel, from left to right are bright filed, fluorescence and merged image of fluorescence and bright field respectively. HTM cells were treated with PM_2.5_ (100 μg/mL) for 48 h. ROS levels were significantly increased in HTM cells treated with Rosup positive control and 100 μg/ml PM_2.5_. Magnification, 10X. **g**. PM_2.5_ exposure impacted on HTM cell contraction. HTM cells were treated with PM_2.5_ (100 μg/mL) for 48 h. The cell contractility was measured after 6 h, 24 h, and 48 h of PM_2.5_ exposure (*n* = 3, *P < 0.05, **P < 0.01, ***P < 0.001, by Student’ t test, compared with the control). Date are represented as the mean ± standard error of mean

To further verify the relationship between PM_2.5_ and pyroptosis in order to explain the effect of PM_2.5_ on IOP, we utilized HTM cells as an in vitro model. As previously mentioned, HTM cells are vital components of outflow tissue, which is the main site of outflow resistance and a key IOP determinant. We initially found that PM_2.5_ was toxic to HTM cells even at 25 μg/mL. When HTM cells were exposed to different PM_2.5_ concentrations for 48 h, a concentration-dependent decrease of cell viability was observed. HTM cell viability was reduced by 18, 37, 37, 42, and 48% after treatment with concentrations of 25, 50, 100, 200, and 400 μg/mL PM_2.5_, respectively (*n* = 5 cell lines, *P* ≤ 0.05, vs. controls groups, Fig. 2a). Similarly, HTM cell viability was reduced by 8, 17, 25, 23, and 37% after treatment with concentrations of 25, 50, 100, 200, and 400 μg/mL PM_2.5_ from Shanghai, respectively (*n* = 5 cell lines, P ≤ 0.05, vs. controls groups, Fig. S1A).

We further observed that NLRP3 protein levels were upregulated 2.7- and 1.6-fold after HTM cells were treated with 100 and 200 μg /mL of PM_2.5_, respectively, for 48 h (*n* = 4 cell lines, *P* < 0.05, Fig. 2b). Quantitative real-time polymerase chain reaction (RT-qPCR) results revealed that 48 h of PM_2.5_ exposure increased the relative expression of NLRP3 mRNA 1.3- and 1.4-fold in 100 μg/mL and 200 μg/mL PM_2.5_-treated HTM cells (*n* = 3 cell lines, *P* < 0.05, Fig. S2A), respectively.

Moreover, the protein expression of caspase-1 increased by factors of 2.3 and 2.6 in PM_2.5_-treated HTM cells compared to controls (100 μg/mL and 200 μg/mL, *n* = 3 cell lines, *P* < 0.01, Fig. 2b). However, the relative expression of caspase-1 mRNA decreased 0.8-fold in PM_2.5_-treated cells (100 μg/mL or 200 μg/mL, *n* = 3 cell lines, *P* < 0.05, Fig. S2A).

Activation of caspase-1 facilitates cleavage of GSDMD, release of the N-terminal GSDMD fragment, and maturation of IL-1β, leading to inflammatory cell death [13]. Notably, in control cell supernatant, the average protein concentration of IL-1β was 12.13 ± 2.56 pg/mL, whereas in PM_2.5_-treated cell culture supernatant, IL-1β levels of 20.42 ± 5.02 and 19.23 ± 5.06 pg/mL were observed after 100 and 200 μg/mL PM_2.5_ treatment, respectively (*n* = 8 cell lines, *P* < 0.05, Fig. 2b). IL-1β mRNA expression was also higher in PM_2.5_-treated HTM cells compared with controls by factors of 15.0 and 15.4 for treatment concentrations of 100 and 200 μg/mL, respectively (*n* = 6 cell lines, *P* < 0.05, Fig. S2A). The protein expression of GSDMD increased by factors of 1.4 and 1.2 in PM_2.5_-treated HTM cells compared to controls (100 μg/mL and 200 μg/mL, *n* = 3 cell lines, *P* > 0.05, Fig. 2b).

To further characterize PM_2.5_-induced pyroptosis of HTM cells, we performed immunofluorescence staining for NLRP3 and caspase-1 after 100 μg/mL PM_2.5_ exposure for 48 h. As shown in Fig. 2d and e, NLRP3 and caspase-1 protein levels increased in PM_2.5_-exposed HTM cells. PM samples from a different location (Shanghai) were also used to test the above-mentioned molecules, which confirmed increased expressions of NLRP3 and caspase-1 by 1.3-fold and 1.0-fold in 100 μg/mL PM_2.5_-treated HTM cells and 2.8-fold and 2.4-fold in 200 μg/mL PM_2.5_-treated HTM cells, respectively (*n* = 3 cell lines, *P* < 0.05, Fig. S1B). GSDMD was upregulated by 1.4-fold, but was not statistically significant which may be due to the small sample sizes (*n* = 3 cell lines, *P* > 0.05, Fig. S1B).

Taken together, these observations revealed the cellular and molecular mechanisms through which PM_2.5_ increased IOP. PM_2.5_ caused cellular toxicity and pyroptosis by activating NLRP3, caspase-1, and GSDMD. This suggests that pyroptosis is an important mediator of ocular cell damage and the decreased cell viability induced by PM_2.5_.

### Increased ROS production and enhanced HTM cell contraction following PM_2.5_ exposure

Prior studies demonstrated that ROS elevation is essential for inflammasome activation [4, 14]. Therefore, in order to verify the role of ROS in PM_2.5_-induced pyroptosis, we measured the changes in intracellular ROS levels in HTM cells after treatment with PM_2.5_ from different locations (100 μg/mL) for 48 h. Representative micrographs of the DCF fluoresce-labeled cells indicate that the PM_2.5_ from different locations elevated ROS production in HTM cells (Fig. 2f, S1C). Furthermore, the contractility of PM_2.5_-treated HTM cells increased 2.4-fold compared to controls after 48 h of treatment (*n* = 3, *P* < 0.05, Fig. 2g), probably due to the increase in ROS levels. Combined with the results presented in Fig. 2, it can be inferred that PM_2.5_-related ROS production likely reduced cell viability and caused pyroptosis. However, further pharmacological experiments are required to test this hypothesis.

### Role of ROS and caspase-1 in PM_2.5_-induced HTM cell pyroptosis

**Fig. 3:**
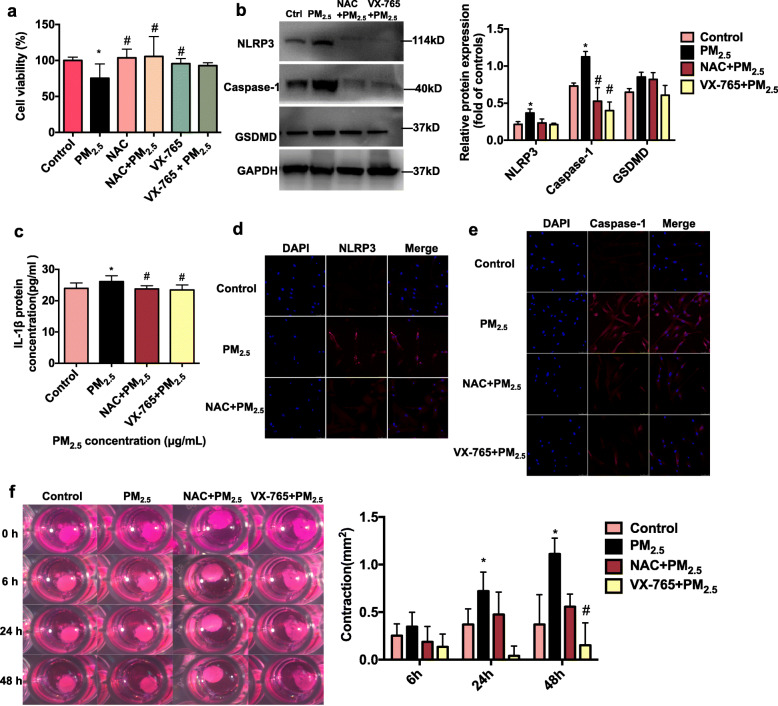
Drug interventions on PM_2.5_ induced toxicity. **a** NAC or VX-765 pretreated for 2 h improved HTM cell viability in cells exposed to 100 μg/mL PM_2.5_ for 48 h (*n* = 5 cell lines, *P < 0.05, one-way ANOVA, compared with the control; #*P* < 0.05, one-way ANOVA, Compare with PM_2.5_ 100 μg/mL). Date are represented as the mean ± standard deviation. **b**, **c** NAC and VX-765 inhibited PM_2.5_ induced NLRP3 inflammasome activation. Relative protein expressions of NLRP3 (*n* = 3), caspase-1 (*n* = 3), GSDMD (*n* = 3) and IL-1β (*n* = 5) were determined by western blot (B) and ELISA (C). *P < 0.05, one-way ANOVA, compared with the control; #*p* < 0.05, one-way ANOVA, compare with PM_2.5_ 100 μg/mL. Ctrl, PBS-treated control; PM_2.5_, PM_2.5_ 100 μg/mL; NAC + PM_2.5_, NAC 3 mM pretreated for 2 h + PM_2.5_ 100 μg/mL; VX-765 + PM_2.5_, VX-765 100 uM pretreated for 2 h + PM_2.5_100 μg/mL. Date are represented as the mean ± standard deviation. **d**, **e** Immunofluorescence examination confirmed that NAC and VX-765 inhibited PM_2.5_ triggered NLRP3 axis activation in HTM cells. Representative immunofluorescence images of the punctate staining of NLRP3 (D) and caspase-1 (E) in HTM cells treated with the vehicle control or PM_2.5_ 100 μg/mL for 48 h, pretreated with or without NAC 3 mM or VX-765 100 μM for 2 h. Blue, DAPI; red, NLRP3 (D) or caspase-1 (E). Magnification, 20X. **f**. NAC and VX-765 inhibited PM_2.5_ induced HTM cell contraction. NAC 3 mM or VX-765 100 μM pretreated for 2 h resulted in a strong inhibition of PM_2.5_ induced HTM contraction. Control, *n* = 3; PM_2.5_, *n* = 4; NAC + PM_2.5_, *n* = 4; VX-765 + PM_2.5_, *n* = 4. *P < 0.05, one-way ANOVA, compared with the control; #*P* < 0.05, one-way ANOVA, compared with PM_2.5_ 100 μg/mL. Date are represented as the mean ± standard error of mean

﻿To further assess if PM_2.5_-induced pyroptosis of HTM cells is triggered by ROS and dependent on caspase-1, we conducted ROS and caspase-1 inhibitory experiments. The ROS scavenger N-acetyl-L-cysteine (NAC) and the caspase-1 selective inhibitor belnacasan (VX-765) were tested for their potential in preventing/alleviating PM_2.5_-induced cell damage. Figure 3a reveals that PM_2.5_ exposure (100 μg/mL) for 48 h lowered HTM cell viability by 25% (*n* = 5 cell lines, *P* < 0.05), while pretreatment with NAC (3 mM) markedly increased cell viability by 40% (*n* = 5, *P* < 0.05). Furthermore, NAC (3 mM) pretreatment reduced ROS production in HTM cells treated with PM_2.5_ from different locations (Fig. S1C, S2B). Similarly, after VX-765 (100 μM) pretreatment, the viability of HTM cells exposed to PM_2.5_ increased by 23% (*n* = 5, *P* > 0.05). Cell viability was similar in HTM cells treated with NAC, VX-765, and the drug vehicle (Fig. 3a, Fig. S2C). Consistent with these results, NAC and VX-765 significantly inhibited PM_2.5_-induced HTM cell contraction (Fig. 3f). These results suggest that the ROS scavenger and caspase-1 inhibitor may help prevent PM_2.5_-induced cell damage.

Our results further demonstrated that NAC and VX-765 significantly downregulated the expressions of the NLRP3, caspase-1, GSDMD, and IL-1β (Fig. 3b and c). Figure 3d and e showed that NAC inhibited NLRP3 and caspase-1 expressions in PM_2.5_-exposed HTM cells, and a distinct decrease in caspase-1 was observed in the HTM cells treated with VX-765 after PM_2.5_ exposure (Fig. 3e).

## Discussion

This is the first study to demonstrate that PM_2.5_ exposure leads to ocular hypertension and glaucoma by inducing cell pyroptosis and inflammation in intraocular tissues responsible for controlling IOP. Reducing ROS production or inhibiting caspase-1 prevented PM_2.5_-induced inflammation and pyroptosis of HTM cells.

Various eye diseases such as dry eye syndrome, conjunctivitis, and keratitis are attributed to air pollution, especially PM_2.5_ pollution, according to ﻿epidemiological and experimental studies [15–18]. PM_2.5_ exposure is also linked to high blood pressure [19], a condition with a pathological mechanism resembling that of high IOP [20, 21]. For example, oxidative stress promotes vascular aging and damage, which contributes to hypertension [22]. Similarly, oxidative stress induces TM cell damage, which impairs aqueous humor drainage, thereby causing ocular hypertension [23]. However, PM_2.5_ has not yet been reported to induce ocular hypertension. Jama et al., however, suggested that environmental black carbon exposure may represent a risk factor for increased IOP in individuals susceptible to other biological oxidative stressors [7]. Consistent with previous studies [7–9], we observed that topical application of PM_2.5_ suspension (1 mg/mL) can cause ocular hypertension in mice (Fig. 1b). To the best of our knowledge, the ﻿relationship between PM_2.5_ and IOP-related disease was unproven in vivo, and this study is the first to report the association between PM_2.5_ and IOP changes in mice.

The classical pyroptosis pathway is central to PM_2.5_-induced injury. Previous studies have reported PM_2.5_-induced NLRP3 inflammasome activation and ocular injury in vivo and in vitro [16, 24–32]. The NLRP3 inflammasome is a vital component of sterile- and infection-triggered inflammation as well as of the immune responses to various diseases [33]. Classical pyroptosis is mediated by the NLRP3 inflammasome, and caspase-1 activation promotes the cleavage of pro-IL-1β and GSDMD [34]. Previous studies have revealed that NLRP3 inflammasome activation greatly contributes to cardiovascular, neurological, and lung disease development [26–32]. In addition, ROS has been reported to activate the NLRP3 inflammasome in environment-induced dry eye syndrome and conjunctivitis, and a significant increase in inflammatory factors, such as IL-1β, was observed [15–18]. However, whether the NLRP3 inflammasome participates in PM-induced injury in outflow tissues has been poorly studied. Consistent with previous studies, our results revealed an increase in NLRP3 protein levels in outflow tissues of PM_2.5_ topically-treated mouse eyes (Fig. 1c). We further demonstrated the PM_2.5_-induced upregulation of the caspase-1, which indicates NLRP3 inflammasome activation. Increased IL-1β protein and cleaved IL-1β were also observed (Fig. 1c). In addition, most of the PM used in the experiments was less than 2.5 μm in size by DLS detection (Fig. 1a) which was consistent with fluorescent PM_2.5_ tracer experiments that some PM_2.5_ could cross the cornea and entered the eye (Fig. 1d). These findings suggest that the NLRP3/caspase-1/IL-1β axis is active in PM_2.5_-induced ocular hypertension. Since TM tissue is vital for the regulation of IOP, and their damage is closely associated with increased aqueous outflow resistance and IOP elevation [35], we suggest that HTM cells undergo pyroptosis during PM_2.5_-induced ocular hypertension.

TM cells are involved in IOP regulation, and PM_2.5_ reduced the viability of the HTM cells by triggering cell pyroptosis. It has been shown that the malfunction of HTM ﻿induced by oxidative stress or vascular damage increases IOP [36]. PM_2.5_ carries toxic components, such as metal, nitrate, sulfate and polycyclic aromatic hydrocarbons (PAHs), and a biological fraction (such as endotoxin), and is known to induce cell death in various ways, including autophagy and pyroptosis [37, 38]. In our study, PM_2.5_-induced pyroptosis and cell dysfunction were observed in HTM cells. The PM_2.5_ used in our study is rich in PAHs and was collected from Lanzhou [39]. When HTM cells were exposed to different PM_2.5_ concentrations (25–400 μg/mL) for 48 h, cell viability was reduced in a concentration-dependent manner (Fig. 2a, Fig. S1A). PM_2.5_ toxicity was also observed in PM_2.5_-exposed or diesel exhaust particle-treated human umbilical vein endothelial cells, (HUVECs), hippocampal neuron cells, bronchial epithelium cells, cornea, and conjunctiva human cell lines [4, 25, 38, 40–42]. According to the published studies [38, 43], PM_2.5_ concentrations from 50 μg/mL to 10 mg/mL have been used in other cells types, therefore, we selected 100 μg/mL and 200 μg/mL of PM_2.5_ to investigate the effect of PM_2.5_ in in vitro toxicology experiments. We further found that PM_2.5_ exposure increased the expressions of NLRP3, caspase-1, IL-1β, and GSDMD proteins in HTM cells (Fig. 2b-e, Fig. S1B). These findings were consistent with the observations in the in vivo experiments (Fig. 1) and were further verified in the next rescue experiments.

ROS production can trigger NLRP3 inflammasome-associated proteins production and inflammatory responses [4], and high ROS levels are detrimental to HTM cells and have been linked to ocular hypertension in glaucoma studies [35, 44]. In our study, a significant elevation of ROS was observed in HTM cells treated with 100 μg/mL PM_2.5_ for 48 h (Fig. 2f, Fig. S1C), along with enhanced HTM cell contractility (Fig. 2g). HTM contractility is an important regulator of conductivity and decreases cell permeability and aqueous humor outflow by reducing the size of intercellular spaces, thereby promoting IOP elevation. In contrast, cell relaxation has the opposite effects [45]. The current observations indicate that oxidative stress damage induces HTM cell dysfunction through NLRP3-mediated pyroptosis.

NAC is a well-known ROS scavenger that decreases ROS production [46], while VX-765 is an effective selective inhibitor of caspase-1 with potent anti-inflammatory activity through inhibition of IL-1β and IL-18 release [47]. Previous studies also reported that NAC can decrease ROS levels and downregulated NLRP3 expression in HUVECs treated with cooking oil fume-derived PM_2.5_ [25]. Nicotine-induced atherosclerosis via ROS/NLRP3-mediated endothelial cell pyroptosis is also prevented by NAC and VX-765 [48]. In our study, NAC and VX-765 were used to further verify the PM_2.5_-related HTM cell injury mechanism. PM_2.5_ at a concentration of 100 μg/mL was employed for further mechanistic experiments. Consistent with other studies, our results revealed that NAC (3 mM) or VX-765 (100 μM) pretreated for 2 h improved HTM cell viability following PM_2.5_ exposure (Fig. 3a). NAC pretreatment efficiently reduced ROS levels and HTM contraction associated with PM_2.5_ exposure (Fig. S1C, S2B, Fig. 3f), inhibiting NLRP3, caspase-1, IL-1β, and GSDMD activation (Fig. 3b-e). VX-765 pretreatment also resulted in the relaxation of HTM cells following PM_2.5_-induced contraction (Fig. 3f) and inhibited caspase-1-mediated pyroptosis (Fig. 3b-e). Hence, these results indicate that the PM_2.5_ exposure elevated oxidative stress, which partially enhanced HTM cell contraction and contributed to an increase in IOP and activation of NLRP3/caspase-1/IL-1β signaling. These in vitro observations supported the results from experiments in mice where elevated IOP was observed following PM_2.5_ exposure, in parallel to oxidative stress damage and NLRP3/caspase-1-mediated pyroptosis. Together with the results in Fig. 2, our data suggest that PM_2.5_-induced ROS production further triggered HTM cell pyroptosis, which is the underlying mechanism of ocular hypertension. NAC and VX-765 might be viable protective compounds against PM_2.5_-induced ocular hypertension.

This study used glass fibre filters (without resin binding) to collect PM samples. Glass filters are non-hygroscopic and are extremely difficult to ash by chemicals or heat thus are increasingly used for PM sampling. A previous study reported a loss of filter mass after PM extraction and the blank filters control caused an increase in cytokine release from macrophages. In our study, we did not observe any visible breakage or particle dissociation from the glass fibre filters during the PM extraction process. There was a slight change in the filter weights before PM collection and after a standard extractions procedure used in our study (Supplement Table S1). Nevertheless, the glass fibre toxicity on TM cells was evaluated (Fig. S3A). Assuming 5–30% glass fibre contamination, a dose-response relationship was established which showed little cytotoxicity. NLRP3 and caspase-1 expression in TM cells was not affected by 30% glass fibre contamination (Fig. S3B). This is consistent with what has been reported using human mesothelial cells, in that, NLRP3 inflammasome cannot be activated by glass beads [49].

Topical applications of PM_2.5_ (1 mg/mL) caused corneal injury ﻿according to our previous study as well as other in vivo studies [43, 50, 51]. The concentration was 5–10 times higher than the concentration used for in vitro experiments. It was estimated that less than 5% of the drops can enter the anterior chamber [52]. Assuming 5% of the drops enter the anterior chamber, each application of PM_2.5_ delivers 0.1 μg PM_2.5_ into the anterior chamber. And in this study, PM_2.5_ was applied three times a day for 3 months.

There are several limitations to our study. We were not able to collect sufficient PM_2.5_ samples for fine particulate matter components analysis. At the same location, our group analysed PM_2.5_ components and found elevated amounts of PAHs [39]. Future studies should investigate the effect of major PM_2.5_ components and the role of each one in the pathological process of PM_2.5_-induced ocular hypertension. Furthermore, we were not able to calculate the exposure concentration from the particle deposition rate for the study period. The PM_2.5_ level ranges from 46 to 163 μg/m^3^ (average level 42.8 μg/m^3^, Fig. S4), which is markedly higher than the nation’s air quality standards (35 μg/m^3^) according to the Environment Protection Agency. However, the PM_2.5_ exposure concentrations used in this study are consistent with the common concentration range in the literature [50, 53, 54], which has been calculated based on the particle deposition rate [54]. For cell culture experiments, cytoxicity was observed with 500 μg/mL PM_2.5_ [54] and 1 mg/mL PM_2.5_, which caused ocular surface damage in rats [50]. Material and assay interference was not measured in the study. However, in each experiment, a stringent control is included and we showed, using cell viability as an example, that materials used had little interference on the results. HTM cells were treated with culture medium, medium plus PBS, and medium plus DMSO and PBS, no difference in cell viability was observed (Fig. S2C).

## Conclusion

This study provides novel evidence that PM_2.5_ has a direct toxic effect on the intraocular tissues and may contribute to the initiation and development of ocular hypertension and glaucoma. This occurs as a result of increased oxidative stress and the subsequent induction of NLRP3 inflammasome mediated pyroptosis in TM cells.

## Materials and methods

### Airborne particulate matter (PM_2.5_) collection

Atmospheric PM_2.5_ samples were obtained from May 2016 to Dec 2018 in Lanzhou, China, and from Jan 2018 to Dec 2019 in Shanghai, China, according to our previously described methods [39]. PM samples were gathered on glass fibre filters (APFF04700, Millipore, USA) by a flow air particle sampler (TH-150C, Wuhan Tianhong Instrument Factory, Wuhan, China) at a constant flow rate of 100 L/min. The filter membranes were then cut into 1 cm × 1 cm squares and extracted three times using an ultrasonic extractor at 100 W for 15 min in deionized water. Next, each sample suspension was filtered using 12 gauze layers, and dried by a vacuum freeze-drying machine (Labconco, Kansas, USA). PM_2.5_ samples were stored at − 80 °C, ﻿followed by resuspension of the resulting pellets in phosphate-buffered saline (PBS) before use.

### Dynamic light-scattering (DLS)

The size distribution of the PM_2.5_ suspended in pure water was analyzed using a Nano-Zetasizer (Malvern Instruments Ltd., Worcestershire, UK) based on the dynamic light-scattering measurement technique, and the sample was first ultrasonicated with water (100 W, 15 min), then 1 ml sample was put into the measuring instrument start to start the measurement.

### Animals and topical exposure of PM_2.5_ suspension

C57BL/6 mice (3–4 weeks old) were purchased from the Shanghai Sippr-BK Laboratory Animal Co. Ltd. Mice were housed in clear cages loosely covered with air filters and containing a corncob pad as bedding. After a week of acclimatization, mice were exposed to 1 mg/mL PM_2.5_ suspension thrice daily (3 × 2 μL drops) for 3 months, with PBS applied topically to the contralateral eye as a control. After 3 months, the mice were sacrificed and outflow tissues were isolated and collected for western blot analysis. All experiments complied with the Association for Research in Vision and Ophthalmology Statement for the use of animals for ophthalmic and vision research and were performed under the guidance of the Animal Care and Use Committee of Fudan University (Shanghai, China).

### PM_2.5_ distribution

The fluorescent mock PM_2.5_ particles prepared from SiO_2_ (diameter: 10–500 nm) were a gift from Prof. Yonghui Deng at Fudan University. Particles were dissolved in PBS and sonication was performed to disperse SiO_2_ particles. Then, the fluorescent particles in PBS were topically applied to the eyes of mice. Frozen sections were prepared, and particle distributions were observed under a confocal fluorescence microscope (Leica, Shanghai, China).

### Mice IOP measurements

The IOP for both eyes was measured﻿ without anaesthesia by rebound tonometry (TonoLab; ICare, Espoo, Finland). IOP was measured three times, and the average was used as the final value.

### Western blotting

Mouse outflow tissue and HTM cells were lysed using a RIPA solution (Beyotime, Shanghai, China), and the protein concentration was determined using the BCA method (Beyotime). Approximately 5–20 μg of protein were loaded onto gels and separated by SDS-PAGE (10% or 12% acrylamide). Proteins were then transferred onto polyvinylidene fluoride membranes (PVDF, 0.45 μm; Millipore, Shanghai, China) by electrophoresis. Membranes were blocked with 5% non-fat dry milk for 2 h at room temperature and probed with a primary antibody (dilutions of the primary antibodies are presented in Table 1), followed by incubation with peroxidase-conjugated secondary antibodies. Glyceraldehyde 3-phosphate dehydrogenase (GAPDH) was used as a loading control.

**Table 1:** Primary antibodies used in the study

Antibody	Source	Catalog No	Type of Ab	Dilution	MW
NLRP3	Abcam	ab214185	Rabbit polyclonal	1:1000 (WB) 1:200 (IF)	114 kD
Caspase-1	Abcam	ab207802	Rabbit polyclonal	1:1000 (WB)	35, 70 kD
Caspase-1	Proteintech	22,915–1-AP	Rabbit polyclonal	1:200 (IF)	
IL-1β	Abcam	ab9722	Rabbit polyclonal	1:1000 (WB)	30, 45 kD
GSDMD	Abcam	ab215203	Rabbit monoclonal	1:1000 (WB)	35, 250 kD
GAPDH	Abcam	ab8245	Mouse monoclonal	1:1000 (WB)	36 kD

*CST* Cell Signaling Technology, *IF* immunofluorescence, *MW* molecular weight, *WB* western blotting

### HTM cell culture and PM_2.5_ treatment

HTM cells were purchased from ScienCell Research Laboratories (Shanghai, China). HTM cells were incubated in Trabecular Meshwork Cell Medium (ScienCell, Cat. No. 6591) containing 2% foetal bovine serum (FBS, Cat. No. 0010), 1% HTM growth supplement (TMCGS, Cat. No. 6592), and 1% penicillin/streptomycin (P/S, Cat. No. 0503) at 37 °C and 5% CO_2_.

For the experiments, HTM cells were seeded at a concentration of 5 × 10^5^ cells/well in 6-well plates and 5 × 10^3^ or 5 × 10^6^ cells/well in 96-well plates. After cell attachment, the culture medium was replaced with a fresh medium containing a PM_2.5_ suspension or an equal volume of medium as a control. NAC (Sigma, Shanghai, China) 3 mM or VX-765 (Selleck, Shanghai, China) 100 μM were used. NAC and VX-765 were first dissolved in DMSO (10,010,023, Gibco, Shanghai, China) and stored at − 20 °C. For rescue experiments, NA and VX-765 were diluted with culture medium (NAC: 3 mM; VX-765: 100 μM). All experiments were performed at least three times.

### Cell viability test

The viability of PM_2.5_-treated HTM cells was tested using a cell counting kit-8 (CCK-8) assay (Dojindo, Kumamoto, Japan) according to the manufacturer’s instructions. Briefly, 100 μL of HTM suspensions (5000 cells/well) were added into the wells of a 96-well plate. After cell attachment, the culture medium was replaced with different concentrations of PM_2.5_ suspension (25, 50, 100, 200, and 400 μg/mL) diluted with fresh medium or an equal volume of medium as a control. After a 48-h incubation, CCK-8 solution (1:10) was added to each well of the plate and cultured at 37 °C for 2 h. Then, the optical density (OD) was measured at 450 nm using a microplate reader (Tecan, Männedorf, Switzerland), and cell viability was reported as a percentage of the OD values from unexposed control cells (100%). In NAC or VX-765 rescue experiments, NAC (3 mM) or VX-765 (100 μM) was applied 2 h before PM_2.5_ exposure. And cell viability of HTM cells treated with culture medium, medium plus PBS, and medium plus DMSO and PBS were tested to find out whether the materials we used had interference on the results.

### Contractility assay and treatments

Collagen gels were prepared in 96-well plates from a collagen solution (1.85 mg/mL, Cell Biolabs, Beijing, China) by following the manufacturer’s instructions. Briefly, HTM (4 × 10^6^ cells/mL medium) was added onto the collagen gel and incubated for 1 h at 37 °C with 5% CO_2_. After collagen polymerization, the culture medium was added to each collagen gel lattice. Following a 48-h incubation, the edge of the gel was gently detached using a pipette tip. The gel area was then imaged using a Fluorescent Stereomicroscope (M165 FC, Leica) every hour for 15 h to determine the time required for cessation of the “natural contraction” of the gel by HTM cells. Drugs (NAC (3 mM) or VX-765 (100 μM)) were added to the medium, and images were captured at 6, 24, and 48 h. The gel area was calculated using the Fluorescent Stereomicroscope (M165 FC, Leica, China).

### ROS production detection

The intracellular ROS levels were detected using the Reactive Oxygen Species Assay Kit (ROS Assay Kit), following the manufacturer’s instructions (Beyotime). After 100 μg/mL PM_2.5_ exposure for 48 h, HTM cells were washed with DMEM/F12 without FBS and then treated with 2′,7′-dichlorodihydrofluorescein diacetate (DCFH-DA, 1:1000, diluted with DMEM/F12) at 37 °C for 20 min. After washing three times with the medium without FBS, the DCF fluorescence distribution of cells was detected. Rosup 50 μg/mL and 500 μg/mL were employed as the negative and positive controls, respectively. The DCF fluorescence distribution of cells was observed under a fluorescence microscope (ZEISS, Shanghai, China).

### RNA isolation and RT-qPCR

HTM cells were exposed to PM_2.5_ (100 μg/mL and 200 μg/mL) for 48 h, while control cells were treated with PBS. Total RNA was then extracted using an RNeasy Mini Kit (Qiagen, Valencia, CA, USA), and RNA concentration was measured using a NanoDrop 2000 spectrophotometer (Thermo Scientific, Wilmington, DE, USA). mRNA expression was measured using the SYBR Green quantitative real-time PCR kit (Takara, Osaka, Japan) according to the manufacturer’s instructions. Samples were amplified in a ViiA 7 Real-Time PCR System (Life Technologies, Pleasanton, CA, USA), and mRNA expression was normalized to β-actin (the housekeeping gene). Expression was estimated using the comparative CT method (2^-ΔΔCT^) of relative quantification using the ViiA 7 Software (Life Technologies). Three independent experiments were conducted. The primer sequences used for the RT-qPCR are presented in Table 2.

**Table 2:** Primers used for RT-PCR

Gene	Primer
NLRP3	Forward: 5′-GCACTTGCTGGACCATCCTC-3’
	Reverse: 5′-GTCCAGTGCACACGATCCAG-3’
Caspase-1	Forward: 5′-AAGACCCGAGCTTTGATTGACTC-3’
	Reverse: 5′-AAATCTCTGCCGACTTTTGTTTCC-3’
IL-1β	Forward: 5′-TATTACAGTGGCAATGAGG-3’
	Reverse: 5′-ATGAAGGGAAAGAAGGTG-3’
β-actin	Forward: 5′-CCCTGGACTTCGAGCAAGAG − 3’
	Reverse: 5′-TCACACTTCATGATGGAGTTG-3’

### Enzyme-linked immunosorbent assay (ELISA)

The IL-1β protein levels in the HTM cell culture medium were quantified using the human IL-1β ELISA kit (Abcam, Boston, MA, USA) following the manufacturer’s instructions. Briefly, 100 μL of each standard and sample were added into appropriate wells and cover well and incubated for 2.5 h at room temperature. Then, the solution was discarded, washed four times, 100 μL of 1× Biotinylated IL-1 beta Detection Antibody was added to each well and incubated for 1 h at room temperature with gentle shaking. Next, the solution was discarded, washed four times, 100 μL of 1× HRP-Streptavidin solution was added to each well and incubated for 45 min at room temperature with gentle shaking. After that, the solution was discarded, washed four times, 100 μL of TMB One-Step Substrate Reagent was added to each well and incubated for 30 min at room temperature in the dark with gentle shaking. Finally, 50 μL of Stop Solution was added to each well and the OD was immediately read at 450 nm using a microplate reader (Tecan).

### Immunofluorescence

Immunofluorescence analysis was performed using specific primary antibodies against NLRP3 (Abcam) and caspase-1 (ProteinTech, Shanghai, China). HTM cells grown on coverslips were fixed in 4% paraformaldehyde for 30 min at room temperature and washed three times with PBS. Then, the cells were treated with 0.1% Triton X-100 (Biotech Well, Shanghai, China) in PBS for 10 min and once again washed three times with PBS. This was followed by blocking in PBS containing 0.5% bovine serum albumin (BSA, Roche, Shanghai, China) for 1 h at room temperature in a humidified chamber. The coverslips were then incubated with a primary antibody (dilutions of the primary antibodies are presented in Table 1) diluted in PBS containing 0.5% BSA overnight at 4 °C in a humidified chamber. The cells were then washed three times with PBS and incubated with Alexa Fluor®555 anti-rabbit IgG (H + L) (1:200; donkey polyclonal; Beyotime) for 1 h at room temperature. After further washing with PBS, coverslips were stained with DAPI and stored at 4 °C in the dark before being viewed under a confocal fluorescence microscope (Sp8, Leica).

### Statistics

The results are presented as the mean ± standard deviation (SD) or mean ± standard error of mean (SEM). Data were analyzed using SPSS 21.0 (IBM, Chicago, IL, USA). For normally distributed data, the paired t-test, independent t-test, or the Student’s t-test were used for two-level comparisons, while one-way analysis of variance (ANOVA) was used for ≥3-level comparison. The Mann-Whitney U test was used for two-level comparisons, while the Kruskal-Wallis H test was used for ≥3-level comparisons of non-normally distributed data. In all cases, differences were considered significant at *P* < 0.05.

## Supplementary Information

**Additional file 1: Fig. S1.** A. Cytoxicity of the PM_2.5_ on HTM cell. For the PM_2.5_ collected in Shanghai between Jan 2018 to Dec 2019, cell viability was analyzed by CCK-8 assay. The human trabecular meshwork cells were treated with different concentrations of Shanghai PM_2.5_ (0 μg/mL, 25 μg/mL, 25 μg/mL, 50 μg/mL, 100 μg/mL, 200 μg/mL, or 400 μg/mL) from Shanghai for 48 h (*n* = 5 cell lines, *P < 0.05, **P < 0.01, ***P < 0.001, by one-way ANOVA, compared with the control). Date are represented as the mean ± standard deviation. **B**. Expressions of NLRP3 inflammasome-related proteins exposed to PM_2.5_ collected in Shanghai. After HTM cells were treated by PM_2.5_ 100 μg/mL or 200 μg/mL, relative protein expressions of NLRP3 (*n* = 3), caspase-1 (*n* = 3), and GSDMD (*n* = 3) determined by western blot (*P < 0.05, one-way ANOVA, compared with the control). Date are represented as the mean ± standard deviation. **C**. ROS production in HTM cells exposed to the PM_2.5_ collected in Shanghai. Representative ROS fluorescence staining images of Rosup positive control (PC, 500 μg/mL), PBS control group (control), PM_2.5_ 100 μg/mL group (PM_2.5_) and NAC + PM_2.5_ group. ROS levels were detected by Reactive Oxygen Species Assay Kit. In each panel, from left to right are bright filed, fluorescence, and merged images of fluorescence and bright field. HTMs were treated with Shanghai PM_2.5_ 100 μg/mL for 48 h, pretreated with or without NAC 3 mM for 2 h. ROS levels were significantly increased in HTMs treated with Rosup positive control and 100 μg/ml PM_2.5._ Magnification, 5X. **Fig. S2. A**. Expressions of NLRP3 inflammasome-related mRNA in PM_2.5_-treated TM cells. qPCR showed mRNA expression of NLRP3 increased significantly after HTMs treated by PM_2.5_ 100 μg/mL or 200 μg/mL (*n* = 3 cell lines, *P < 0.05, one-way ANOVA, compared with the control). The mRNA expression of caspase-1 decreased after HTMs treated by PM_2.5_ 100 μg/mL or 200 μg/mL (*n* = 3 cell lines, *P < 0.05, one-way ANOVA, compared with the control). The mRNA expression of IL-1β increased significantly after HTMs treated by PM_2.5_ 100 μg/mL or 200 μg/mL (*n* = 6 cell lines, *P < 0.05, one-way ANOVA, compared with the control). **B**. NAC inhibited ROS elevation in Lanzhou PM_2.5_-treated HTM cells. Representative ROS fluorescence staining images of Rosup negative control (NC, 50 μg/mL), Rosup positive control (PC, 500 μg/mL), PBS control group (control), PM_2.5_ 100 μg/mL group (PM_2.5_) and NAC + PM_2.5_ group. ROS levels were detected by Reactive Oxygen Species Assay Kit. In each panel, from left to right are bright filed, fluorescence, and merged images of fluorescence and bright field. ﻿HTMs were treated with PM_2.5_ 100 μg/mL for 48 h, pretreated with or without NAC 3 mM for 2 h. ROS levels were significantly increased in HTMs treated with Rosup positive control and 100 μg/ml PM_2.5._ Magnification, 10X. **C**, Cytoxicity of the PBS and DMSO on HTM cell. Cell viability was analyzed by CCK-8 assay. The human trabecular meshwork cells were treated with culture medium (control), medium plus PBS, medium plus DMSO and PBS, respectively according to our used dose in the study (*n* = 5 cell lines, *p* > 0.05, by one-way ANOVA, compared with the control). Date are represented as the mean ± standard deviation. **Fig. S3. A**. Effects of fiber-exposure on HTM cell viability. Cell viability were analyzed by cell viability assay (CCK-8). The human trabecular meshwork cells were treated with different concentrations of fiber (0 μg/mL, 5 μg/mL, 20 μg/mL, 30 μg/mL) for 48 h. (*n* = 6 cell lines, *P < 0.05, by one-way ANOVA, compared with the control). Date are represented as the mean ± standard deviation. **B**. NLRP3 inflammasome-related protein expressions in glass fibre treated HTM cells. After HTM cells were treated by 30 μg/mL fibers obtained from glass fibre filters that were used for PM collection. Relative protein expressions of NLRP3 and caspase-1 were determined by western blot (*n* = 3 cell lines, *P* > 0.05, independent t-tests, compared with the control), which did not differ significantly from control cells. Date are represented as the mean ± standard deviation. **Fig. S4. A**. Air quality index (AQI) in Lanzhou between May 2016 and Dec 2018. The PM_2.5_ level ranges from 46 to 163 μg/m^3^ with an average level of 42.8 μg/m^3^ from May 2016 to Dec 2018 in Lanzhou. **B.** Air pollutants in Lanzhou between May 2016 and Dec 2018. The figure shows the concentration of PM_2.5_ (μg/m^3^), PM_10_ (μg/m^3^), SO_2_ (μg/m^3^), CO (mg/m^3^), NO_2_ (μg/m^3^), O_3_ (μg/m^3^) in the air from May 2016 to Dec 2018 in Lanzhou. **Table 1** Weight of glass fiber filtres before and after PM extraction.

## Data Availability

The datasets during and/or analysed during the current study available from the corresponding author on reasonable request.

## Abbreviations

PM: Particulate matter
IOP: intraocular pressure
HTM: Human trabecular meshwork
ROS: Reactive oxygen species
TM: Trabecular meshwork
SC: Schlemm’s canal
NAC: N-Acetyl-L-cysteine
VX-765: Belnacasan
CCK-8: Cell counting kit-8
