# Supplementary material for: Airborne particulate matter (PM2.5) triggers ocular hypertension and glaucoma through pyroptosis
Source: Part Fibre Toxicol. 2021 Mar 4;18:10. doi: 10.1186/s12989-021-00403-4 (PMC7934500; doi:10.1186/s12989-021-00403-4)
Supplement: Supplementary file 1 — Additional file 1: Fig. S1. A. Cytoxicity of the PM2.5 on HTM cell. For the PM2.5 collected in Shanghai between Jan 2018 to Dec 2019, cell viability was analyzed by CCK-8 assay. The human trabecular meshwork cells were treated with different concentrations of Shanghai PM2.5 (0 μg/mL, 25 μg/mL, 25 μg/mL, 50 μg/mL, 100 μg/mL, 200 μg/mL, or 400 μg/mL) from Shanghai for 48 h (n = 5 cell lines, *P < 0.05, **P < 0.01, ***P < 0.001, by one-way ANOVA, compared with the control). Date are represented as the mean ± standard deviation. B. Expressions of NLRP3 inflammasome-related proteins exposed to PM2.5 collected in Shanghai. After HTM cells were treated by PM2.5 100 μg/mL or 200 μg/mL, relative protein expressions of NLRP3 (n = 3), caspase-1 (n = 3), and GSDMD (n = 3) determined by western blot (*P < 0.05, one-way ANOVA, compared with the control). Date are represented as the mean ± standard deviation. C. ROS production in HTM cells exposed to the PM2.5 collected in Shanghai. Representative ROS fluorescence staining images of Rosup positive control (PC, 500 μg/mL), PBS control group (control), PM2.5 100 μg/mL group (PM2.5) and NAC + PM2.5 group. ROS levels were detected by Reactive Oxygen Species Assay Kit. In each panel, from left to right are bright filed, fluorescence, and merged images of fluorescence and bright field. HTMs were treated with Shanghai PM2.5 100 μg/mL for 48 h, pretreated with or without NAC 3 mM for 2 h. ROS levels were significantly increased in HTMs treated with Rosup positive control and 100 μg/ml PM2.5. Magnification, 5X. Fig. S2. A. Expressions of NLRP3 inflammasome-related mRNA in PM2.5-treated TM cells. qPCR showed mRNA expression of NLRP3 increased significantly after HTMs treated by PM2.5 100 μg/mL or 200 μg/mL (n = 3 cell lines, *P < 0.05, one-way ANOVA, compared with the control). The mRNA expression of caspase-1 decreased after HTMs treated by PM2.5 100 μg/mL or 200 μg/mL (n = 3 cell lines, *P < 0.05, one-way ANOVA, compared with the control) [file 12989_2021_403_MOESM1_ESM.docx]

**Supplementary Materials**

**Airborne particulate matter (PM_2.5_) triggers ocular hypertension and glaucoma through pyroptosis**

*Liping Li^1*^, Chao Xin*g*^2*^, Ji Zhou^3^, Liangliang Niu^1^, Bin Luo^4,5^ , Maomao Song^1^, Jingping Niu ^3^,* Ye Ruan *^4#^, Xinghuai Sun^1,6,7#^, Yuan Lei^1,6#^*

^1^Department of Ophthalmology & Visual Science, Eye Institute, Eye & ENT Hospital, Shanghai Medical College, Fudan University, Shanghai 200031, China

^2^Experimental Research Center, Eye & ENT Hospital, Shanghai Medical College, Fudan University, Shanghai 200031, China

^3^Shanghai Key Laboratory of Meteorology and health, Shanghai, 200030, China.

^4^Institute of Occupational Health and Environmental Health, School of Public Health, Lanzhou University, Lanzhou 730000, Gansu, China

^5^Shanghai Key Laboratory of Meteorology and Health, Shanghai Meteorological Bureau, Shanghai, China

^6^ NHC Key Laboratory of Myopia, Chinese Academy of Medical Sciences (Fudan University), and Shanghai Key Laboratory of Visual Impairment and Restoration (Fudan University), Shanghai 200031, China

^7^State Key Laboratory of Medical Neurobiology and MOE Frontiers Center for Brain Science，Institutes of Brain Science, Fudan University, Shanghai 200032, China

*Co-first authors

# Correspondence:

Yuan Lei, Department of Ophthalmology & Visual Science, Eye Institute, Eye & ENT Hospital, Shanghai Medical College, Fudan University, Shanghai, China. E-mail: lilian0167@hotmail.com. [yuan_lei@fudan.edu.cn](https://wx.qq.com/cgi-bin/mmwebwx-bin/webwxcheckurl?requrl=http%3A%2F%2Fyuan_lei%40fudan.edu.cn&skey=%40crypt_38773abf_2dc7535a65b1c06f339c13f9e3b86332&deviceid=e367073530633624&pass_ticket=rbEenyCTKsAwn3Ut0eZA6JUHxCQ9VxIVsB7y%252BSfGlfrBTgWyacxsRkQIUliWbiVq&opcode=2&scene=1&username=@30df32478fa573b52308d345c4a2ff124dd26e6cce807e3ff29c319103fc4e31).

Xinghuai Sun, Department of Ophthalmology & Visual Science, Eye Institute, Eye & ENT Hospital, Shanghai Medical College, Fudan University, Shanghai, China. E-mail: xhsun@shmu.edu.cn.

Ye Ruan, Institute of Occupational Health and Environmental Health, School of Public Health, Lanzhou University, Lanzhou 730000, Gansu, China. E-mail: [ruany@lzu.edu.cn](mailto:ruany@lzu.edu.cn).

**
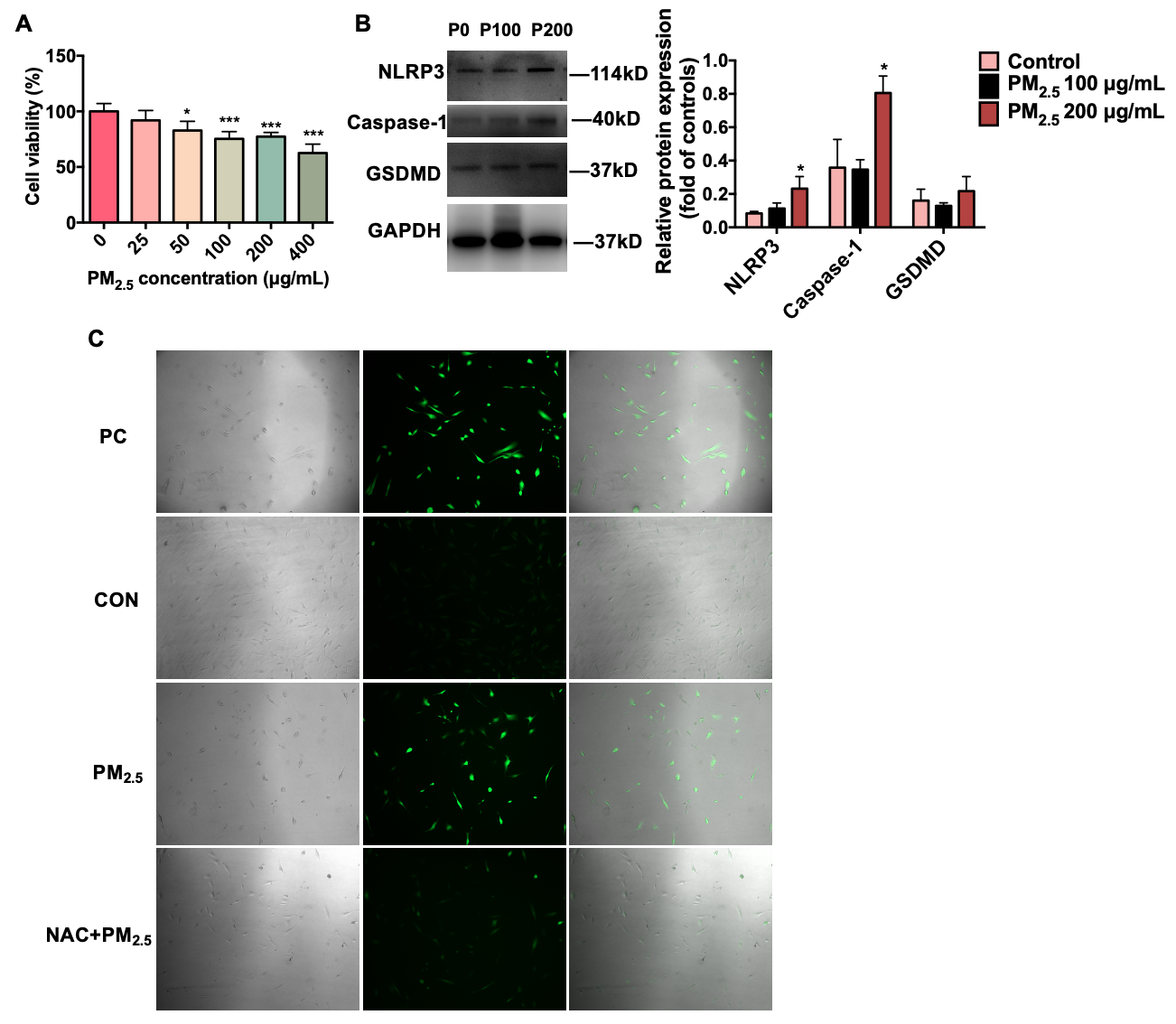
Figure** **S1. A**. Cytoxicity of the PM_2.5_ on HTM cell. For the PM_2.5_ collected in Shanghai between Jan 2018 to Dec 2019, cell viability was analyzed by CCK-8 assay. The human trabecular meshwork cells were treated with different concentrations of Shanghai PM_2.5_ (0 μg/mL, 25 μg/mL, 25 μg/mL, 50 μg/mL, 100 μg/mL, 200 μg/mL, or 400 μg/mL) from Shanghai for 48 h (n = 5 cell lines , *P < 0.05, **P < 0.01, ***P < 0.001, by one-way ANOVA, compared with the control). Date are represented as the mean ± standard deviation. **B**. Expressions of NLRP3 inflammasome-related proteins exposed to PM_2.5_ collected in Shanghai. ﻿After HTM cells were treated by PM_2.5_ 100 μg/mL or 200 μg/mL, relative protein expressions of NLRP3 (n = 3), caspase-1 (n = 3), and GSDMD (n = 3) determined by western blot (*P < 0.05, one-way ANOVA, compared with the control). Date are represented as the mean ± standard deviation. **C**. ROS production in HTM cells exposed to the PM_2.5_ collected in Shanghai. Representative ROS fluorescence staining images of Rosup positive control (PC, 500 μg/mL), PBS control group (control), PM_2.5_ 100 μg/mL group (PM_2.5_) and NAC+PM_2.5_ group. ROS levels were detected by Reactive Oxygen Species Assay Kit. ﻿In each panel, from left to right are bright filed, ﬂuorescence, and merged images of ﬂuorescence and bright field. ﻿HTMs were treated with Shanghai PM_2.5_ 100 μg/mL for 48 h, pretreated with or without NAC 3 mM for 2 h. ROS levels were significantly increased in HTMs treated with Rosup positive control and 100 ug/ml PM_2.5._ Magnification, 5X.

**
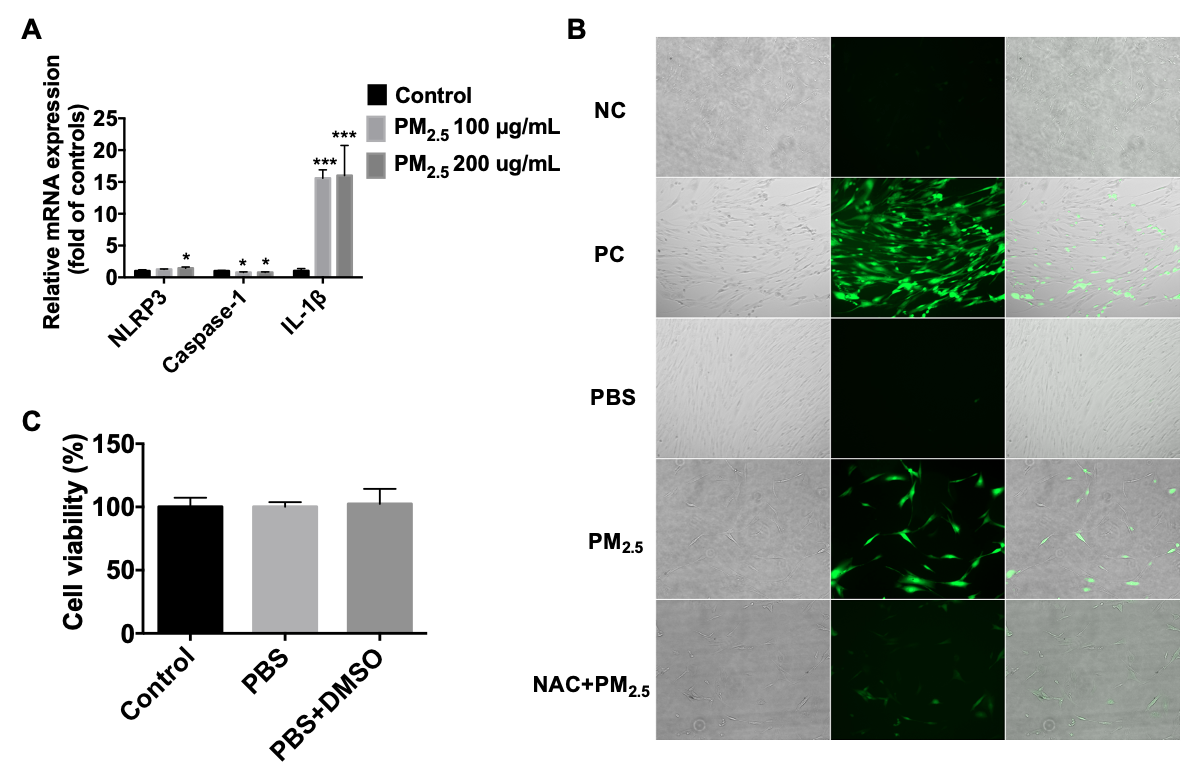
**

**Figure S2. A**. Expressions of NLRP3 inflammasome-related mRNA in PM_2.5_-treated TM cells. qPCR showed mRNA expression of NLRP3 increased significantly after HTMs treated by PM_2.5_ 100 μg/mL or 200 μg/mL (n = 3 cell lines, *P<0.05, one-way ANOVA, compared with the control). The mRNA expression of caspase-1 decreased after HTMs treated by PM_2.5_ 100 μg/mL or 200 μg/mL (n = 3 cell lines, *P<0.05, one-way ANOVA, compared with the control). The mRNA expression of IL-1β increased significantly after HTMs treated by PM_2.5_ 100 μg/mL or 200 μg/mL (n = 6 cell lines, *P<0.05, one-way ANOVA, compared with the control). **B**. NAC inhibited ROS elevation in Lanzhou PM_2.5_-treated HTM cells. Representative ROS fluorescence staining images of Rosup negative control (NC, 50 μg/mL), Rosup positive control (PC, 500 μg/mL), PBS control group (control), PM_2.5_ 100 μg/mL group (PM_2.5_) and NAC+PM_2.5_ group. ROS levels were detected by Reactive Oxygen Species Assay Kit. ﻿In each panel, from left to right are bright filed, ﬂuorescence, and merged images of ﬂuorescence and bright field. ﻿HTMs were treated with PM_2.5_ 100 μg/mL for 48 h, pretreated with or without NAC 3 mM for 2 h. ROS levels were significantly increased in HTMs treated with Rosup positive control and 100 ug/ml PM_2.5._ Magnification, 10X. **C**, Cytoxicity of the PBS and DMSO on HTM cell. Cell viability was analyzed by CCK-8 assay. The human trabecular meshwork cells were treated with culture medium (control), medium plus PBS, medium plus DMSO and PBS, respectively according to our used dose in the study (n = 5 cell lines , p > 0.05, by one-way ANOVA, compared with the control). Date are represented as the mean ± standard deviation.

**
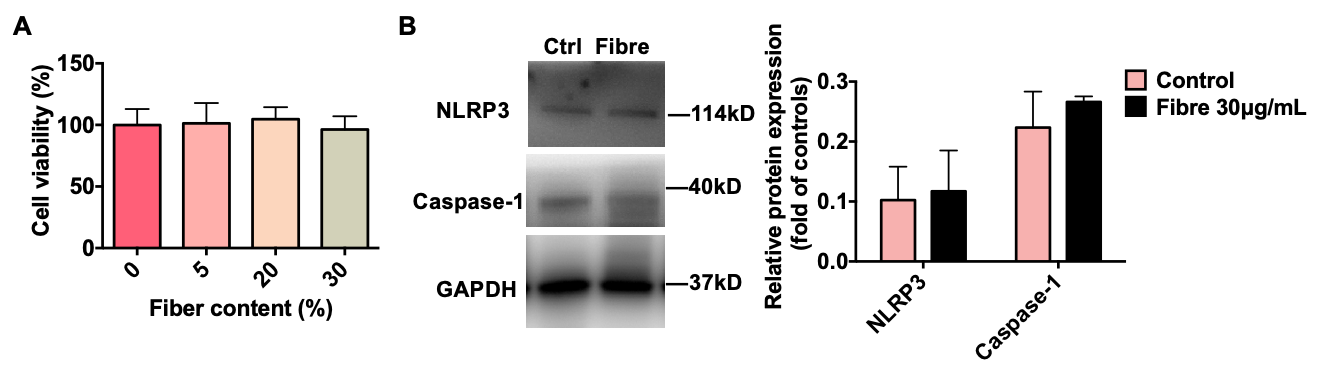
**

**Figure S3. A**. Effects of fiber-exposure on HTM cell viability. Cell viability were analyzed by cell viability assay (CCK-8). The human trabecular meshwork cells were treated with different concentrations of fiber (0 μg/mL, 5 μg/mL, 20 μg/mL, 30 μg/mL) for 48 h. (n = 6 cell lines , *P < 0.05, by one-way ANOVA, compared with the control). Date are represented as the mean ± standard deviation. **B**. NLRP3 inflammasome-related protein expressions in glass fibre treated HTM cells. ﻿After HTM cells were treated by 30 μg/mL fibers obtained from glass fibre filters that were used for PM collection. Relative protein expressions of NLRP3 and caspase-1 were determined by western blot (n = 3 cell lines, P > 0.05, independent t-tests, compared with the control), which did not differ significantly from control cells. Date are represented as the mean ± standard deviation.

**
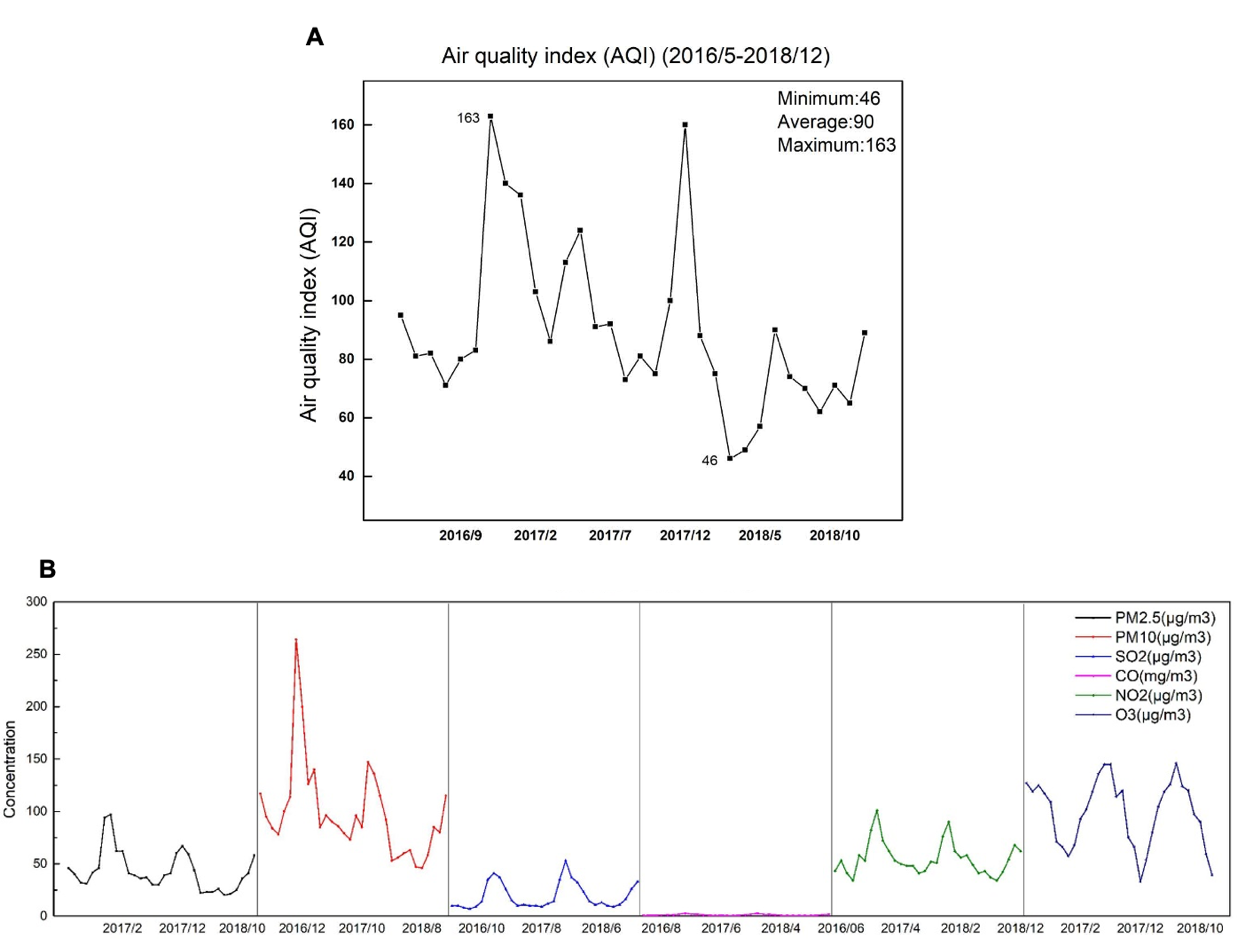
**

**Figure S4. A.** Air quality index (AQI) in Lanzhou between May 2016 and Dec 2018. The PM_2.5_ level ranges from 46 to 163 μg/m^3^ with an average level of 42.8 μg/m^3^ from May 2016 to Dec 2018 in Lanzhou. **B.** Air pollutants in Lanzhou between May 2016 and Dec 2018. The figure shows the concentration of PM_2.5_ (μg/m^3^), PM_10_ (μg/m^3^), SO_2_ (μg/m^3^), CO (mg/m^3^), NO_2_ (μg/m^3^), O_3_ (μg/m^3^) in the air from May 2016 to Dec 2018 in Lanzhou.

| **Table 1 Weight of glass fiber filtres before and after PM extraction** | |
| --- | --- |
| Glass fiber filtre | Weight (g) |
| Before extraction | 0.4679 |
| After extraction | 0.4619 |
| Change of the filtre weights | 0.0060 (1.28%) |
